# https://www.fungiofpakistan.com: a continuously updated online database of fungi in Pakistan

**DOI:** 10.1093/database/baab080

**Published:** 2021-12-29

**Authors:** Mubashar Raza, Lei Cai, Muhammad Waseem Abbasi, Marium Tariq, Nalin N Wijayawardene

**Affiliations:** State Key Laboratory of Mycology, Institute of Microbiology, Chinese Academy of Sciences, Chaoyang District, Beijing 100101, P.R. China; Department of Plant Pathology, College of Agriculture, University of Sargodha, Sargodha, Punjab 40100, Pakistan; State Key Laboratory of Mycology, Institute of Microbiology, Chinese Academy of Sciences, Chaoyang District, Beijing 100101, P.R. China; Department of Botany, University of Karachi, Karachi, Sindh 75270, Pakistan; M.A.H. Qadri Biological Research Centre, University of Karachi, Karachi, Sindh 75270, Pakistan; Center for Yunnan Plateau Biological Resources Protection and Utilization, Qujing Normal University, Qujing, Yunnan 655011, P.R. China

## Abstract

The website fungiofpakistan.com is a collection of all the available data about macro- as well as micro-fungi collected from Pakistan. This website comprises reported fungal species with isolation source or host record, locality and updated classification. The data on this website is based on old literature (library data, personal data of specific authors or books that were not easily accessible to public) and recent publications. This website is an important potential platform for researchers, government officials, industries and other users. Users can provide their inputs related to missing taxa, new genera, the new record and new data. They also have the opportunity to express their opinions on valid names, invalid names and illegitimate names, with notes published in the ‘Notes’ section of webpage provided following review and editing by curators and fungal taxonomists. This website plays a significant contribution to our knowledge of the rich fungal diversity of Pakistan. However, much more sustained and detailed research is needed to fully evaluate fungal diversity in Pakistan. Undoubtedly, that many more fungi will be discovered and added in the future.

**Database URL:**

https://fungiofpakistan.com/

## Introduction

Pakistan is recognized as one of the mega-diverse countries in the world with estimated 6000 plant species known from Pakistan (1, 2). Despite years of research, the flora of Pakistan is poorly known. Generally, the flora of Irano-Turanian Region (Western Himalayan Province) is well known than the other provinces of Pakistan. The diversity of *Asteraceae, Apiaceae, Brassicaceae, Fabaceae* and *Lamiaceae* is known from various published works (3). The information on other families is still lacking and their checklist for Pakistan is yet to be compiled. In contrast, the fungal diversity of Pakistan is much less well known than that of flora of Pakistan.

Identifying this biodiversity gap, back in 1999, Syed Irtifaq Ali (S.I Ali) from University of Karachi (principal editor of flora of Pakistan) proposed a plan to Peter H. Raven from Missouri botanical garden, USA to complete the flora of Pakistan as a co-publisher and efforts are still underway. Likewise, the first introductory chapter about the fungal checklist that contained more fungal species than identified by Sultan Ahmed (S. Ahmed) from the different regions of Pakistan was introduced by Mirza and Qureshi (4). A brief history of collecting fungi in Pakistan has been detailed below. Given limited fungal taxonomic expertise and the resources available at their disposal in the country, it is highly unlikely to obtain a complete inventory of fungi. Experience from numerous other projects that involved expert fungal taxonomist equipped with resources has shown that more concentrated work needs to be conducted over several decades.

Nevertheless, efforts have been underway at the University of the Punjab (PU), since June 2003 wherein they established ‘first Fungal Culture Bank of Pakistan’ (FCBP) to build inventory of the fungi and have published many fungal species in the newsletter (previously known as ‘Myconews’ which recently changed to ‘Agrinews’) or local journal (Mycopath) with accession numbers (5). We collected the information of isolated strains and have put it on a single platform (fungiofpakistan.com). This information could be very useful not only for comprehensive record and better appreciation of Pakistan fungal biodiversity but also as a source of reference for other scientists working on the same aspect of fungi such as biological control, biotechnology, fungi for food and medicine, fungal genetics, pest and disease control, plant pathology and other related subjects.

The fungi, according to updated classification, listed in the fungiofpakistan.com online database are sourced from books, published articles and inventory of FCBP. Presently, our website includes members from nine fungal phyla. These phyla are Ascomycota, Basidiomycota, Blastocladiomycota, Chytridiomycota, Entomophthoromycota, Glomeromycota, Kickxellomycota, Mortierellomycota, Mucoromycota and Zoopagomycota. Fungi-like organism/taxa belong to Hyphochytriomycota and Oomycota (Kingdom Straminipila). Nevertheless, this does not imply that other phyla members are absent in Pakistan. It only demonstrates that they have not been reported in the available literature. We may have left out a few fungi especially those reported in older publications or in publications that we overlooked (in this case, users have an option to submit the data). All fungal genera used in the list have been checked and updated with those listed in the outline of fungi and fungi-like taxa (6), Index Fungorum and/or Mycobank (7) but species name further needs to be checked either are valid names, invalid names or illegitimate names.

## Brief history and previous checklists

Knowledge regarding Fungi of Pakistan endeavor variety of macro- and micro-fungi from different areas of the country. One of the major documents on the fungal history of sub-continent is Butler and Bisby (8) that provided a list of 2351 fungi belonging to order Uredinales, perithecial stages of family Erysiphaceae (15 species) and sooty moulds (64 species) from British India. Seventy collections of Indian smuts were critically re-identified by Mundkur (9). Mundkur (10) studied and listed fungi from North western part of India. However, Butler and Bisby (8) and Mundkur (10) published around 200 species of order Uredinales from the area that represents Pakistan (formally known as West Pakistan before independence of East Pakistan). Then, Mundkur and Ahmad collaborated to work together on the description of different groups of fungi from Pakistan.

The earlier known collections of fungi from the regions that make up present-day Pakistan i.e. West Punjab and Sindh, Punjab and KPK provinces, or some parts of Sindh province including Karachi, were made between 1948 and 1972 by several studies (11–21) and by Ghaffar in late 60s and early 70s (22–24). Many species of coelomycetous fungi from southern parts of Pakistan were collected and described by Sutton and Abbas (25), Abbas and Sutton (26) and Abbas *et al.* (27–31).

Fungal species of order Agaricales, number of rust and smut species were described comprehensively by Ahmad (32), Ono (33) and Ono and Kakishima (34, 35). Myco-flora was also contributed by various authors individually in the form of publications from specified areas instead of any monograph or booklet such as ‘Mushrooms of Kashmir’ (36, 37), Basidiomycota of Kaghan Valley (38), fungi on mangrove plants (39), checklist of the Lichens (40), checklist of *Boletales* (41, 42), checklist of Ascomycetes and Gasteromycetes of Kaghan Valley (43), species diversity in Basidiomycota of district Malakand (44) and records of *Russula* species (45).

The most eminent and influential mycologist in Pakistan was S. Ahmed (Sultan Ahmed). His contribution to botany and conservation is well known and laid the groundwork for understanding of fungal myco-flora and huge taxonomic work for biodiversity in Pakistan. His comprehensive work recorded 1219 species in Pakistan and was published in 1997 (46). The second edition of Fungi of Pakistan was a reprint of the first edition without any updated information, published in 2014 (47). Many of his collections were deposited in Mycological Herbarium of the Department of Botany, University of Punjab, Lahore, Pakistan, duplicates in Herb. I.M.I, Kew, Surrey, England and also in the Mycological Herbarium of USDA, Beltsville, MD, USA. Nothing is known about the fate of those early collections that were deposited in Mycological Herbarium in Pakistan and others, they are now probably all lost or destroyed. How we can trace and find their deposition record is still a question mark as those are not listed in the publications.

Various culture collection centers across the country work for myco-flora isolation, identification and deposition of various culturable strains. The first Fungal Culture Bank of Pakistan (FCBP) was established in 2003 at the University of Punjab (5). Among all, few of them have a proper online catalogue describing the strain’s history, molecular evidence or status of a publication. Some published data is found having no clue about its disposition to any culture center (48, 49). Due to the unavailability of the published strains at collection centers, their viability is doubtful.

## Gaps and limitations of existing data

The authors recognized a few limitations while compiling the checklist.

- The taxonomic/nomenclature status of many fungi listed by S. Ahmed has since not been revised and the list is outdated.
- Most of the old publications and fungal records present in printed form are unavailable to local as well as the international research community.
- Fungal species that were published before 1958 are still valid although the type was not indicated.
- There is no specific number for fungal species reported from Pakistan and information about which isolate has molecular data is also lacking.

## Overcoming limitations of static publications

 The website fungiofpakistan.com is launched to provide a continuously updated list of fungal species that have been reported from Pakistan since 1947. Despite the previously available data, it is essential and need of the day that reported strains should be organized based on the available information. While compiling the checklist for the website, collection of data, putting it into electronic form and updating it according to the recent classification were indeed a challenge. Other related information such as substrate, the location where they were observed and isolated or collected and the related references are provided. Where ‘unknown’ is stated in the online database under achieve and Fungi of Pakistan hierarchy, especially to substrate or location, indicates that relevant information was not provided in the original publication. Fungal species concerning their culture collection accession number were listed such as ‘PU’ (refers to the University of Punjab) culture collection number. This platform provides valuable information about all reported strains from Pakistan. Its applicability will be helpful to get knowledge about myco-flora of country and also will be able to help researchers to find updated taxonomy, history, molecular details, and status of the strains.

## Fungi of Pakistan web page and logo representation

The Fungi of Pakistan online resource has several strong positive features, and its main objectives are to

- Provide the myco-flora of significant and insufficiently known regions and keep a record of it.
- Present the continually updated consensus of fungi classification.
- Provide a platform to introduce the molecular data of previously reported species rather than to describe them as novel species with molecular data.
- Provide details and notes on important changes to the registered users via this platform.
- Provide an opportunity to graduate students, researchers and scholars to add missing data and put suggestions to modify the data with critical comments based on expert opinions.

**Figure 1.**
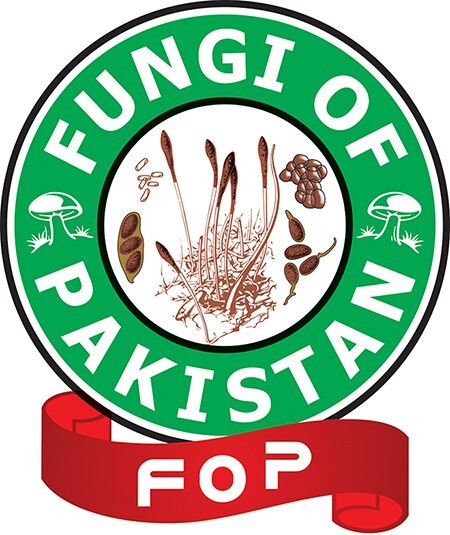
Fungi of Pakistan logo design represents the online database.

Fungi of Pakistan e-portal has a unique logo (Figure 1) and its design represents a clear picture of the online database. The logo is in green color and circular in shape with a red ribbon at the base that includes an abbreviation of Fungi of Pakistan (FOP). Fungal features are brilliantly depicting the array of micro- and macro-fungi within the white and green color scheme. White and green colors are resembled with the flag of Pakistan to present growth, prosperity, purity and uniqueness, while red highlights the passion and love for fungi. The black outline around the green circle is a sign of power, authority, seriousness and strength. The name of the database is featured in a wordmark in the green circle with two macro-fungi on either side while a few characteristics of micro-fungi are featured in the center. In fact, it would not be an exaggeration to say that the unique concept of Fungi of Pakistan logo design will contribute immensely to the website/database’s success.

## Construction

Fungal genera recorded from Pakistan are, listed on the website, following the latest classification of kingdom fungi (6, 50).

## Website interface and visualization

**Figure 2.**
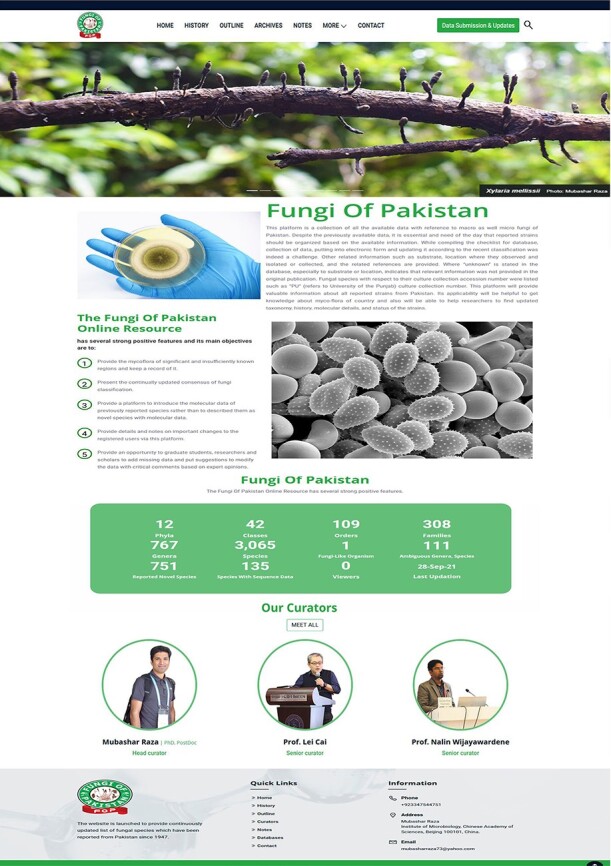
The homepage view of fungiofpakistan.com.

The home page includes seven tabs and other related information including a summary of the online database. We tried our best to make this website user-friendly and simple interface (Figure 2).

## Tools included on the homepage

**Figure 3.**
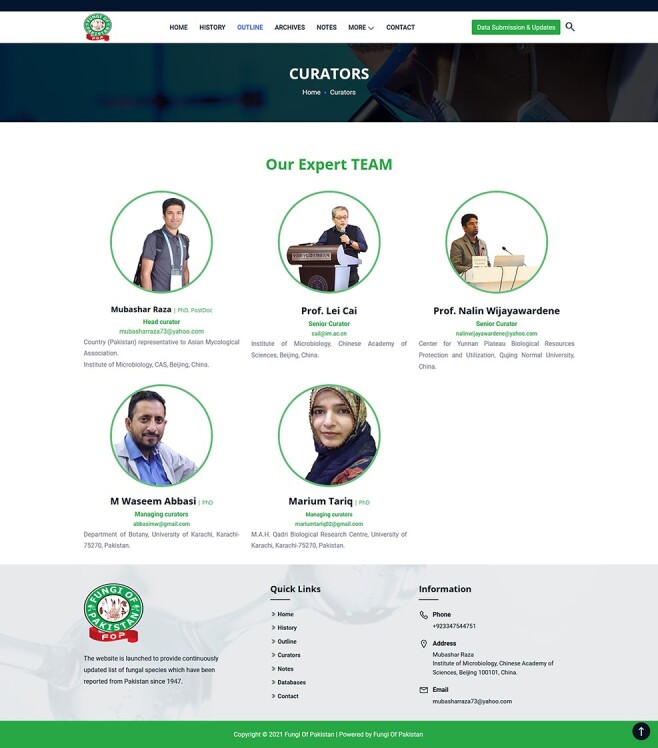
Experts team in database management.

**Figure 4.**
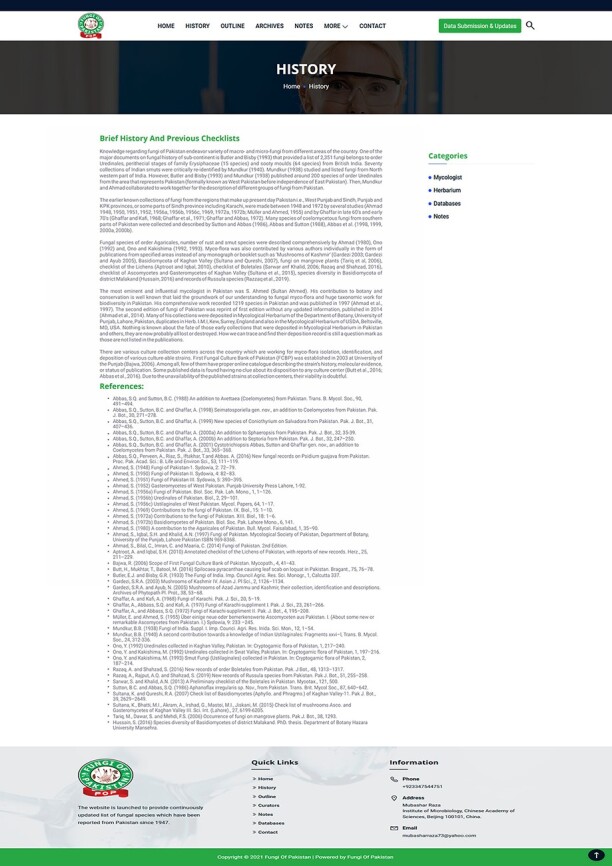
Brief history of fungi collected in Pakistan.

**Figure 5.**
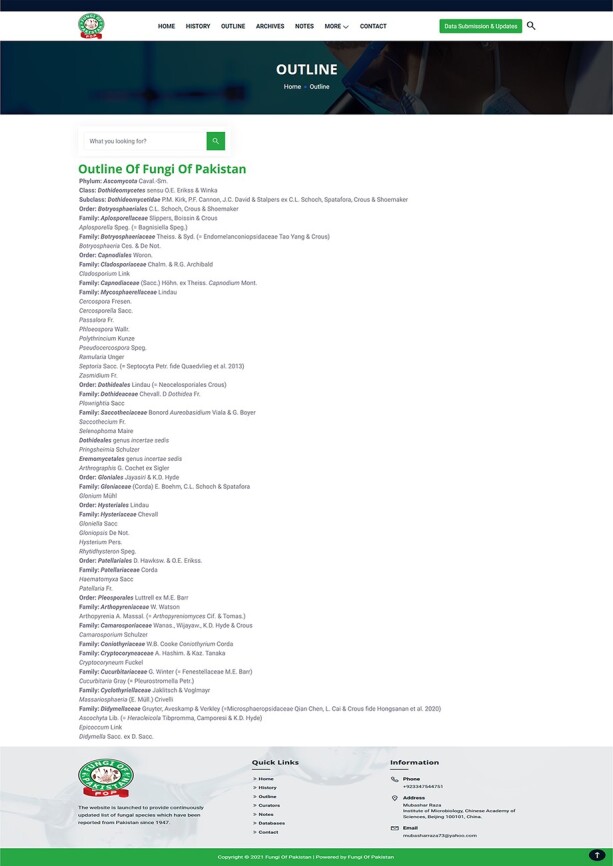
Fungi of Pakistan outline.

**Figure 6.**
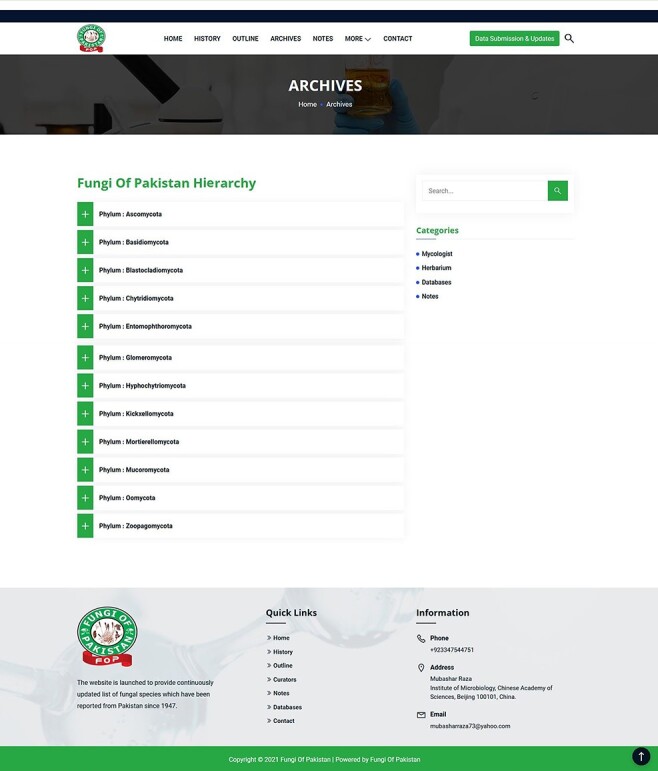
Use of Archives tool.

**Figure 7.**
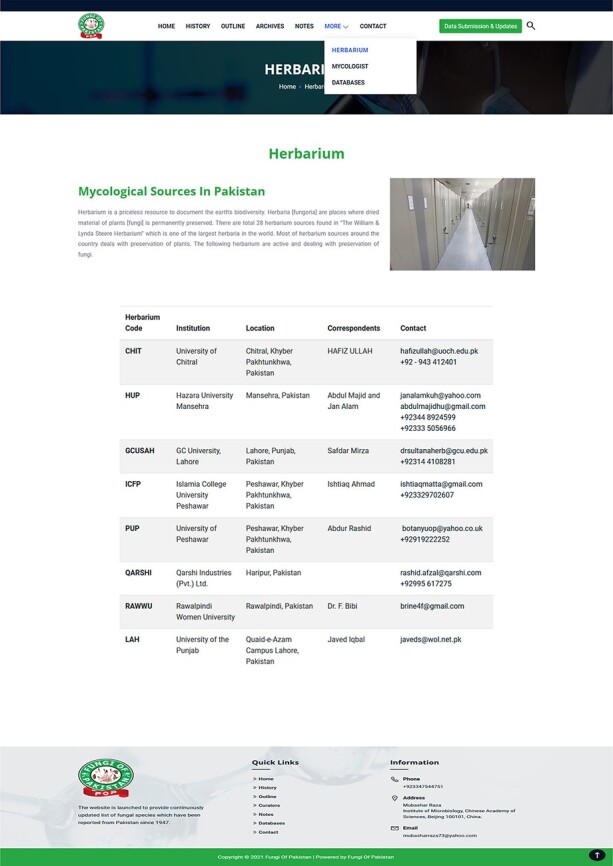
Information on herbarium centers within Pakistan.

**Figure 8.**
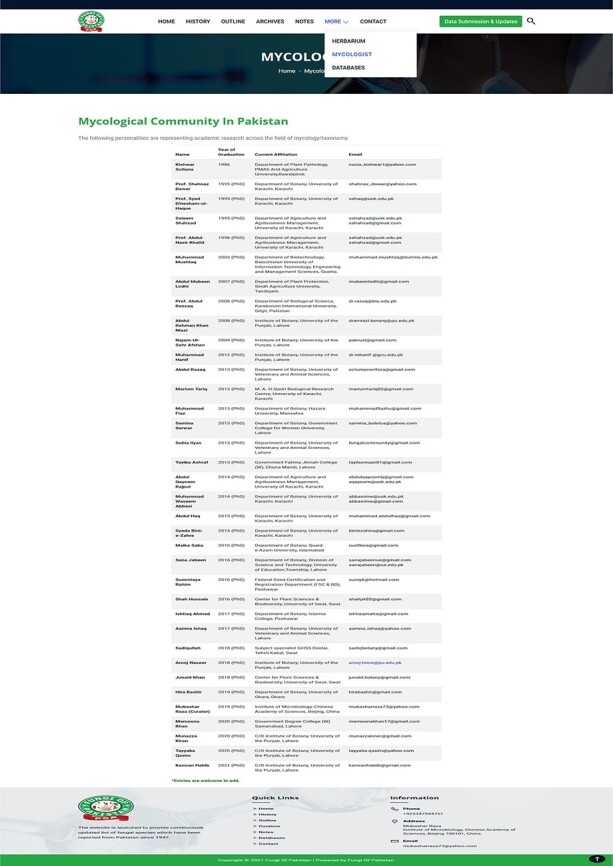
Information of mycological community for international collaboration.

**Figure 9.**
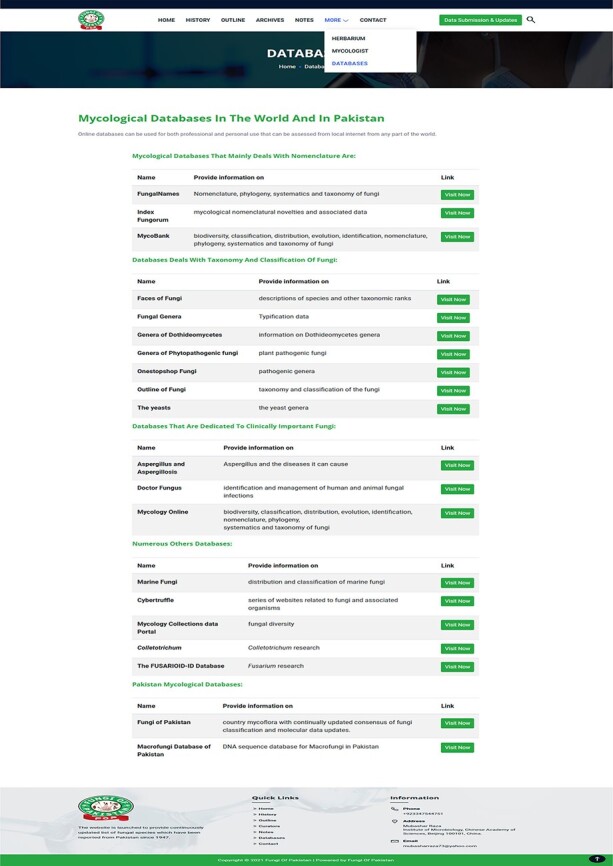
Some fungi related databases.

**Figure 10.**
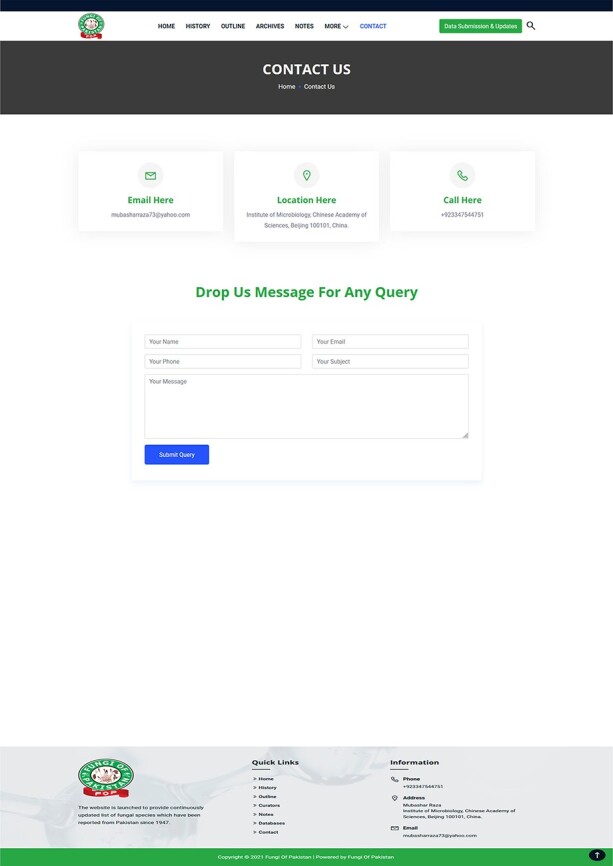
Tool for user comments and suggestions.

The home page includes the following tools:

- Home: This online resource homepage provides an overview of the fungi of Pakistan and the objectives of launching the website. It contains the current number to phyla, classes, orders, families, genus, species, fungal-like taxa, ambiguous genera, reported novel species and reported species with sequence data. It also includes search option, the data submission button and a signup option for the latest updates. The list and contact details of all curators of the website can be found at bottom of the homepage and at the ‘meet all’ option (Figures 2 and 3).
- History: This section provides a brief history of fungi collecting in the region, Pakistan (Figure 4).
- Outline: This section provides the latest classification and list of fungal genera recorded from the region (Figure 5).
- Achieves: This section provides a hierarchy of fungal species reported from Pakistan (Figure 6).
- Notes: This section provides the recent changes in taxonomy, novel species and new records.
- More: This section includes the following options:
  - Herbarium: This part provides the herbarium centers within the country dealing with the preservation of fungal specimens (Figure 7).
  - Mycologists: This part provides the list and contact details of mycologist/taxonomists working in Pakistan (Figure 8).
  - Databases: This part provides the list of online databases related to mycology around the world and in Pakistan (Figure 9).
- Contact: The ‘contact’ section allows the users to address any comment and suggestion (Figure 10).

## Notes section and preparation

This is an important part of fungiofpakistan.com, which provides information on new additions, modifications and user opinions. This provides an opportunity for users to understand recent changes.

Changes could be due to the following main reasons:

- Classification changes by following recent publications.
- Addition of new or missing taxa, reported from Pakistan.
- Corrections and errors in uploaded data (e.g. wrong placement and duplication of taxa).

Preparation of notes will follow specific conditions:

- The addition of new taxa or published material that introduces new taxa is cross-checked with repositories such as Mycobank or Index Fungorum by the managing curators. Their main task is to keep the website up to date. As the second step, the new entries or addition will be checked by the senior curators. Once the new entries are edited by the managing curator, according to the senior comments, the head curator will cross-check the validity of taxa against repositories and upload it to the website. The list of new taxa will also be gathered from MycoBank or Index Fungorum, twice a year. Authors who publish new taxa (from Pakistan) are encouraged to provide entries.
- Notes for missing taxa are expected from website users and expert mycologists. They can use the data submission option on the home page to send the entries to curator (Figure 3).
- Notes that correct errors or mistakes (such as typo errors and incorrect citation) will also be accepted by the website users. However, the head curator will check whether entries are necessary to upload or correct the web version.
